# Measuring health-related quality of life in tuberculosis: a systematic review

**DOI:** 10.1186/1477-7525-7-14

**Published:** 2009-02-18

**Authors:** Na Guo, Fawziah Marra, Carlo A Marra

**Affiliations:** 1 Collaboration for Outcomes Research and Evaluation (CORE), Faculty of Pharmaceutical Sciences, University of British Columbia, Vancouver, B.C., Canada; 2 Faculty of Pharmaceutical Sciences, University of British Columbia; Director, Vaccine and Pharmacy Services, British Columbia Centre for Disease Control (BCCDC), Vancouver, B.C., Canada; 3 Centre for Health Evaluation and Outcome Sciences (CHEOS), Providence Health Care Research Institute, Vancouver, B.C., Canada

## Abstract

**Introduction:**

Tuberculosis remains a major public health problem worldwide. In recent years, increasing efforts have been dedicated to assessing the health-related quality of life experienced by people infected with tuberculosis. The objectives of this study were to better understand the impact of tuberculosis and its treatment on people's quality of life, and to review quality of life instruments used in current tuberculosis research.

**Methods:**

A systematic literature search from 1981 to 2008 was performed through a number of electronic databases as well as a manual search. Eligible studies assessed multi-dimensional quality of life in people with tuberculosis disease or infection using standardized instruments. Results of the included studies were summarized qualitatively.

**Results:**

Twelve original studies met our criteria for inclusion. A wide range of quality of life instruments were involved, and the Short-Form 36 was most commonly used. A validated tuberculosis-specific quality of life instrument was not located. The findings showed that tuberculosis had a substantial and encompassing impact on patients' quality of life. Overall, the anti-tuberculosis treatment had a positive effect of improving patients' quality of life; their physical health tended to recover more quickly than the mental well-being. However, after the patients successfully completed treatment and were microbiologically 'cured', their quality of life remained significantly worse than the general population.

**Conclusion:**

Tuberculosis has substantially adverse impacts on patients' quality of life, which persist after microbiological 'cure'. A variety of instruments were used to assess quality of life in tuberculosis and there has been no well-established tuberculosis-specific instrument, making it difficult to fully understand the impact of the illness.

## Introduction

The assessment of patient reported outcomes (PROs) has become more accepted and valued in the disease management and outcome evaluation. Health-related quality of life (HRQL) is a complex type of PRO that evaluates health status. HRQL broadly describes how well individuals function in daily lives and their own perception of well-being in physical, psychological, and social aspects [1,2]. Although traditional clinical and biological indicators are often intrinsically related to patients' quality of life, they fail to represent one's self-perceived function and well-being in everyday life settings. It is known that patients with chronic diseases place a high value on their mental and social well-being as well as pure physical health [3]. As a result, HRQL has become an area of increasing interest and has been evaluated in many diseases, including tuberculosis (TB). To measure HRQL, two kinds of instruments are often used: generic and disease-specific [1,2,4]. Generic instruments are developed to cover the common and important aspects of health and can be used to assess and compare HRQL across different health conditions and sub-populations [1,4]. In contrast, disease- or condition-specific instruments are designed to reflect unique problems most relevant to a given disease and/or its treatment [1,4]. Theoretically, disease-specific instruments are more precise and more sensitive to small but potentially important differences or changes on HRQL, compared to generic instruments [1,4]. One special category of generic HRQL instruments assesses "preferences" for certain health states [2]. These instruments summarize quality of life into a single utility score, reflecting the 'value' people place on a health state, anchored at 0 (death) and 1 (full health). [2]. Health utility measurements are often used in health economic studies.

Although effective therapy has long been available, TB remains a major public health threat globally, with one third of the world's population infected [5,6]. Many aspects of TB along with its treatment could potentially compromise patients' HRQL. For example, the standard anti-TB therapy consists of four medications and takes at least 6 to 9 months to complete, with serious risks of adverse reactions [6-8]. In some communities, TB patients are perceived as a source of infection and the resultant social rejection and isolation leads to a long-term impairment on patients' psychosocial well-being [9-14]. Many TB patients also report to experience negative emotions, such as anxiety and fear [13,14]. However, the current goal of TB management is to achieve microbiological 'cure' and there has been little effort taken to consider patients' HRQL. In 2004, Chang et. al. published a review summarizing the English medical literature on the quality of life in TB patients [15]. At that time, the authors were unable to locate studies measuring HRQL using standardized instruments. Over the past few years, more effort has been dedicated to this research field. Therefore, the present review was performed to identify published original studies utilizing structured HRQL instruments.

### Objectives

The objectives of this review were: (1) to identify HRQL instruments used in TB research; (2) to better understand the impact of TB disease or infection and the associated treatment on patients' HRQL; and (3) to examine demographic, socio-economic, and clinical factors associated with HRQL outcomes in TB patients.

## Methods

### Search strategies for identification of potential studies

**Figure 1:**
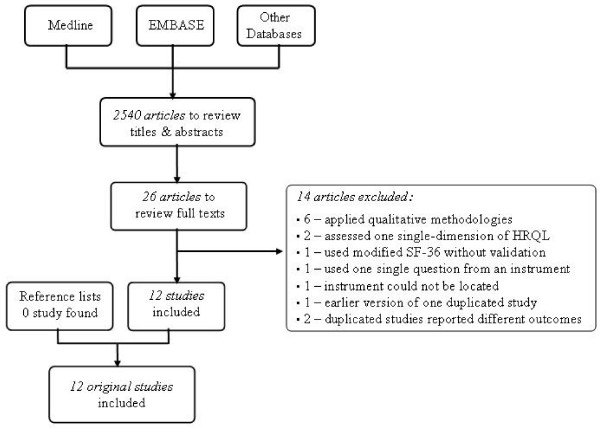


A systematic literature search was performed using the following electronic databases: Medline (1950-present), EMBASE (1980-present), Cochrane Register of Controlled Trials (CENTRAL), CINAHL, PsycINFO, and HaPI (1985-present). Key word searching and/or subject searching were performed, if applicable. The following keywords were used: *tuberculosis (TB), Quality of Life (QoL), Quality Adjusted Life Years (QALY), health utility, health status, life quality*, and *well-being*. The limit feature was used to select human studies published between 1981 and 2008 written in English or Chinese (traditional or simplified). The last time electronic database search was conducted during July 22, 2008. The reference sections of the following key journals were manually searched for relevant articles: *International Journal of Tuberculosis and Lung Disease, Chest, Quality of Life Research*, and *Health and Quality of Life Outcomes*. Reference lists of included studies, review articles, letters, and comments were checked afterwards. We did not contact the authors of identified studies or relevant experts to locate unpublished studies. Each stage of the literature searching process is illustrated in Figure 1.

### Inclusion and exclusion criteria

All clinical trials and observational studies where multi-dimensional HRQL was evaluated, either as a primary or secondary outcome, using structured HRQL instruments were considered in this review. Participants were those diagnosed with active TB disease or latent TB infection (LTBI), regardless of the site and stage of the disease and the treatment status. There were no limitations on age, gender, race, the origin of birth, and other socio-economic status.

For the purpose of this review, HRQL was defined as patients' self-evaluations of the impact of either active TB disease or LTBI and the associated treatments on their physical, mental, and social well-being and functioning. The following requirements for HRQL measurement were set a priori for studies to be included in this review: (1) one multi-dimensional HRQL instrument or a combination of single-dimensional instruments had to be used to capture the broad framework of HRQL; (2) the HRQL instruments could be either generic or disease (or condition) -specific; (3) the origin of the applied instruments had to be identifiable and traceable; (4) the HRQL instruments had to have psychometric properties such as reliability and validity reported from previous studies or were assessed in the specific study being reviewed; (5) HRQL outcomes had to be self-reported by the specific participant, but HRQL measurement that were completed with help from proper proxies, such as family members and caregivers, were also accepted.

Studies were excluded if (1) HRQL was evaluated using qualitative methodologies, such as focus groups; or (2) only one single dimension of HRQL (e.g., depression) was assessed; or (3) HRQL was assessed using instruments designed for the specific study without psychometric properties evaluated and reported; or (4) a modified version of a previously validated instruments (e.g., SF-36) was used as the psychometric properties of the original instrument could be changed by the modification.

### Data extraction

If the study was included in this review, the following information was collected: study design, inclusion and exclusion criteria of subjects, included subjects' socio-demographic characteristics and clinical features, HRQL instrument(s) used, the origin and structure of HRQL instrument(s), administration of HRQL instrument(s), and HRQL outcomes and validation results.

## Results

The literature search identified 2540 articles which were narrowed to 26 [9-14,16-35] (Figure 1). After reviewing the full texts, 14 studies were further excluded for various reasons: 6 studies used qualitative methodologies [9-14]; 2 studies measured only one single dimension of HRQL [16,17]; 1 study [18] used the Short-Form 36 (SF-36) but the response options of SF-36 were modified to 3 levels (i.e., the same as before, better, and worse) without providing validation data; 1 study [19] used one single question from a structured instrument; 1 study was a duplicate and the earlier version was excluded [20,21]; 1 study [22] used a generic instrument, the General Quality of Life Interview (GQOLI-74), however, no relevant references were provided to track the origin and the psychometric properties of this instrument; 2 articles [23,24] were published from the same study, and therefore only included as one study for the review; another 2 articles, Marra et. al. [25] and Guo et. al. [26], reported longitudinal and cross-sectional results from one same study respectively, and thus only one study was counted for the review. Therefore, a total of 12 original studies were included in this review [21,23,25,27-35] and an overview is presented in Additional file 1.

Of the 12 included studies, one was published in 1998 [27] and the remaining 11 were published after 2001 [21,23,25,28-35]. Nine studies were published in English and 3 in Chinese [27,29,33]. The included studies were carried out within different countries: 3 in China [27-29]; 1 in both China and southern Thailand [33]; 2 in India [21,35]; 2 in Turkey [30,31]; 2 in Canada [23-26]; and 2 in the USA [32,34]. Seven of the included studies were cross-sectional [27,29-31,33-35] and 4 were prospective cohort studies [21,23,25,28]. The remaining one study was a randomized controlled trial (RCT) [32], but only baseline HRQL assessment data was reported in the published article. Among the 12 studies, three studies included a comparison group either from the general population [28] or from a "healthy" non-TB sample [27,29]; one study used the normative data from the Canadian population as the reference group [23,24]; two studies included people with LTBI as controls [25,34]; one study compared TB patients with a group of chronic obstructive pulmonary disease (COPD) patients [31]; and the remaining 5 studies did not include proper comparison groups. Sample size (i.e., number of subjects included in the statistical analysis) varied among the 12 studies, from 46 to 436. Only one study [23] reported how the sample size was estimated statistically. A wide range of TB patients were included in this review: pulmonary TB and extra-pulmonary TB, active TB disease and LTBI, and current TB and previously treated TB.

To measure multiple-dimensional HRQL, a variety of instruments were involved in the included studies (Additional file 2). As a result, it was not possible to statistically summarize the results and thus a qualitative synthesis approach was taken for this review.

### HRQL instruments used in the included studies

Nine studies included generic multi-dimensional instruments with or without specific single-dimensional ones, one study used a newly developed TB-specific multi-dimensional instrument [21], and two studies used a battery of single-dimensional instruments [31,33].

#### Generic HRQL instruments

The SF-36 was used in 6 studies with different language versions [23-28,33]. It consists of 36 items which are aggregated into 8 subscales, including physical functioning (PF), role-physical (RP), bodily pain (BP), general health (GH), vitality (VT), social functioning (SF), role-emotional (RE), and mental health (MH) [36]. From the 8 subscales, the physical component summary (PCS) and mental component summary (MCS) scores can be also calculated [36]. Duyan et. al. used the 24-item Quality of Life Questionnaire (QLQ), which covers 7 domains, including living conditions, finances, leisure, family relations, social life, health, and access to health care [30]. The 24-item QLQ was first presented by Greenley et. al. in 1997 [37]. Finally, the long Medical Outcome Study (MOS) core questionnaire was used in Pasipanodya et. al. [34]. This is a generic instrument covering multiple dimensions, including physical function, social function, general health, vitality, and limitations due to physical and emotional functioning [38]. The well-known SF-36 was developed and evolved based on a subset of items from the MOS core questionnaire [38].

#### Specific HRQL instruments

Dhingra and Rajpal measured HRQL with the DR-12, a new TB-specific instrument, which was developed in India and first published in 2003 [20]. It is composed of 12 items, among which 7 cover TB symptoms (i.e., cough and sputum, haemoptysis, fever, breathlessness, chest pain, anorexia, and weight loss) and 5 relate to socio-psychological and exercise adaptation (i.e., emotional symptoms/depression, interest in work, household activities, exercise activities, and social activities) [20,21]. All response options are presented on 3-point scales and equal weights are given to each item when calculating the two domain scores and the total score [20,21]. The St. George Respiratory Questionnaire (SGRQ) used in Pasipanodya et. al. [34] is a widely used specific instrument designed for measuring HRQL in patients with chronic obstructive pulmonary disease (COPD) and other types of respiratory diseases. Three domain (symptom, activity, and impacts) scores and a total score can be generated [39]. It was developed at the St. George's Hospital Medical School at the UK and has been translated into various languages [39].

Yang et. al. used two single-dimensional instruments, the Chinese version Symptoms Checklist 90 (SCL-90) and Social Support Rating Scale (SSRS) [29]. The SCL-90 is a 90-item symptom inventory designed mainly to evaluate a broad range of psychological problems and symptoms, including 9 dimensions: somatization, obsessive-compulsive behaviour, interpersonal sensitivity, depression, anxiety, hostility, phobic anxiety, paranoid ideation, and psychoticism [40]. The 10-item SSRS was used to measure the self-perceived availability and use of social support services [27]. The study by Aydin and Ulusahin used two single-dimensional instruments, the General Health Questionnaire 12 (GHQ-12) and Brief Disability Questionnaire (BDQ) [31]. GHQ-12 is a short version of the GHQ-60, which was developed for screening non-psychotic psychiatric disorders in the general population [41]. The BDQ, derived from the MOS short form general health survey, is used to measure patients' physical and social disability level [42]. Marra et. al. [25] used the Beck Depression Inventory (Beck-DI), along with the SF-36 and a couple of health utility instruments. The Beck-DI is a 21-item instrument, designed to measure the symptoms and degree of depression [43].

In the USA study, a series of instruments or questions were used to assess TB-infected homeless individuals' self-perceived physical health, psychological profile, emotional well-being, social support, and health care access and use [32]. Examples included the 5-item Mental Health Index (MHI-5) and the Center for Epidemiological Studies Depression Scale (CES-D) [32].

#### Health utility instrument

Health utility, one generic measure of HRQL, reflects subjective preferences for health states and also provides quantitative estimates of HRQL under certain health states [2]. The two studies [23-26] conducted in Canada applied various health utility assessment techniques among TB patients, including the Health Utility Index (HUI), EuroQol (EQ-5D), Short-Form 6D (SF-6D), Visual Analogue Scale (VAS), and Standard Gamble (SG). HUI, SF-6D, and EQ-5D are multi-attribute health status classification systems that indirectly measure preferences for health states [2]. SG and VAS are to directly obtain individuals' preferences using different techniques.

HUI currently consists of HUI-2 and HUI-3 [44]. HUI-2 and HUI-3 are derived from the same questionnaire but HUI-2 has 7 domains (sensation, mobility, emotion, cognition, self-care, pain, and fertility) and HUI-3 contains 8 domains (vision, hearing, speech, ambulation, dexterity, emotion, cognition, and pain). EQ-5D consists of 5 domains, including mobility, self-care, usual activity, pain, and anxiety/depression [2]. SF-6D is derived from a subset of SF-36 questions. It has 6 dimensions including physical functioning, role limitations, social functioning, pain, mental health, and vitality [45].

The SG is a classic technique to obtain individual preferences for health outcomes, based on the theory of von Neumann and Morgenstern [2]. In the study by Dion et. al., the respondent was offered a choice between the certain outcome of a particular health state and a hypothetical gamble, with relative possibilities of perfect health and immediate death varying. The gamble was terminated when the respondent was indifferent to the choice between the given health state and the gamble. The VAS used by Dion et. al. was a 100 cm "feeling thermometer", marked at each end by word descriptions as "immediate death" and "perfect health". The respondents were asked to put a mark at the point that represents their current health status [23,24]. Similarly, a 10 cm length of horizontal line (anchored at 0 cm = death and 10 cm = perfect health) was used by Marra et. al. [25] as VAS.

### Psychometric properties of HRQL instruments in tuberculosis

The SF-36 was used in 6 studies, and overall it showed acceptable validity and reliability. Chamla [28] validated the Chinese version SF-36 among active pulmonary TB patients and the general population in China. The reliability was tested by Cronbach's α, ranging form 0.88 to 0.97 for the eight SF-36 subscales. All 36 questions of the SF-36 had internal item consistency coefficients between 0.56 and 0.86. In Dion et. al. [23,24], the reliability of SF-36 was evaluated among a mixture of TB patients, including 25 with LTBI, 17 with active TB on treatment, and 8 with previously treated TB. The internal consistency of the SF-36 responses was strong, with coefficients of 0.86–0.92 for the two summary scores and 0.73–0.94 for the subscale scores. The test-retest reliability (2-week interval) of SF-36 was tested by calculating Intraclass Correlation (ICC) coefficients: 0.66–0.79 for the two SF-36 summary scores. He et. al. [33] also reported good reliability of the Chinese version SF-36 (Cronbach' α > 0.7) among the two groups of TB patients from China and Thailand.

Validity of the SF-36 was evaluated by examining the correlations between SF-36 outcomes with other external variables, including clinical criteria, responses from other HRQL measures, and physician's evaluations. It was reported that SF-36 scores were able to discriminate between TB patients with different severity levels [21,26] and between patients at different stages of treatment (i.e., the start, middle, and end of the treatment) [21,25,28]. In Guo et. al. [26], the correlations between SF-36 summary scores (PCS and MCS) and four utility instruments (SF-6D, HUI-2, HUI-3, and VAS) were tested by calculating Spearman's coefficients. SF-6D scores were strongly correlated with both PCS and MCS (0.79, 0.80), and HUI-2, HUI-3, and VAS scores were more strongly correlated with PCS (0.59, 0.66, and 0.67) than with MCS (0.37, 0.48, and 0.59). Similarly, in the study by Dion et. al. [23,24], SF-36 scores were observed moderately correlated with EQ-5D and VAS scores, but poorly correlated with SG scores (Pearson coefficients < 0.2). Wang et. al. [27] reported that patient-reported SF-36 scores were well correlated with physician proxy-reported Quality of Life Index (QLI) and Karnofsky Performance Status (KPS) scores, with correlation coefficients of 0.78 and 0.89 respectively. However, it was not reported which type of correlation coefficient was calculated.

The structural validity of SF-36 was tested in two studies, but the results were not consistent. In Chamala [28], factor analysis was applied to evaluate the 2-dimensional model of the SF-36. Two factors (physical health and mental health) were extracted and subjected to orthogonal rotation using the Varimax method. The observed pattern of correlations between the 8 subscales and the 2 factors supported the authors' prior hypothesis. For example, it was reported that the 4 physical subscales (PF, RP, BP, and GH) were correlated strongly with the physical health factor, but only poorly correlated with the mental health factor. On the other hand, the 4 mental subscales (MH, RE, SF, and VT) were strongly correlated with the mental health factor, but not the physical factor. He et. al. [33] used principle component analysis to test the structural validity of SF-36. However, the results showed that the 8 subscales were not well independent, and there were overlapping items between different subscales. For example, RE and RP subscales were both strongly correlated among the two groups of patients (correlation coefficient 0.82 and 0.77). Based on their findings, the authors concluded that the SF-36 did not show satisfactory construct validity in the studied TB patients.

The application of SF-36 among TB patients also revealed some problems. In the study by Dion et. al. [23,24], SF-36 subscales demonstrated a remarkable ceiling effect problem. Over 50% participants with concurrent or previous TB reported the highest scores for 5 of SF-36 subscales (PF, RP, RE, BP, and SF).

Ceiling and floor effects are a common problem for the application of health utility instruments in TB. In Dion et. al. [23,24], 42–53% participants reported the best possible EQ-5D health state. Guo et. al. also observed ceiling and/or floor effect problems with three commonly used health utility instruments. HUI-2 and HUI-3 suffered from a serious ceiling effect problem, both in global score and single dimension level. For example, 25% of active TB patients scored 1.0 (perfect health) using the HUI-2 and 98% of them reported the best level of hearing for HUI-3. SF-6D, on the other hand, was primarily limited by its narrow range of available utility values, from 0.30 to 1.0. Health states at the lower end may not be adequately represented by the SF-6D. Despite these problems with the application among TB patients, some positive aspects of these utility instruments were also observed. For example, these utility instruments showed moderate to strong correlations with the SF-36 responses as stated before [23,24,26]. Guo et. al. [26] also reported moderate to strong agreement among SF-6D, HUI-2, HUI-3, and VAS, using ICC: the overall ICC coefficient among these 4 instruments was 0.65 and paired ICC coefficients ranged from 0.53 to 0.67. In addition, these four utility instruments were all able to discriminate between TB patients with different severity levels.

Pasipanodya et. al. [34] administered the lung disease-specific SGRQ among people with treated pulmonary TB disease or LTBI. Test-retest reliability of the SGRQ was examined by ICC coefficients, 0.93 for the total score and 0.83–0.91 for subscale scores. Internal consistency was tested by Cronbach's α, at 0.93. To evaluate its validity, SGRQ responses were correlated with a previously validated MOS core questionnaire and a couple of clinical pulmonary function tests, such as the forced vital capacity (FVC). Overall, SGRQ scores and MOS scores agreed on similar health constructs and diverged on dissimilar constructs. Low but significant correlations were observed between SGRQ scores and pulmonary function test results (-0.12 to -0.29, p < 0.05). On the other hand, a ceiling effect problem for SGRQ was observed. In both treated pulmonary TB patients and people with LTBI, the distribution of SGRQ scores was skewed toward higher HRQL. In addition, considering varied levels of reading and understanding in English in respondents, different language versions of SGRQ were used, but the potential impact of combining results from these on HRQL outcomes was not known.

Dhingra and Rajpal [21] applied the new TB-specific instrument, DR-12, among TB patients under directly observed therapy (DOT). It was reported that, at the beginning of treatment, DR-12 scores demonstrated significant differences between pulmonary and extra-pulmonary TB patients, and between sputum positive and sputum negative patients. Over the treatment period, higher DR-12 score gains were observed among patients who positively responded to the treatment compared to those who did not. Based on these evidences, the authors came to the conclusion that DR-12 had strong construct validity in the studied population. However, the clinical criteria or indicators were not well defined in the published work. All comparisons were performed by using paired or unpaired t-tests. Potential confounders such as socio-demographic and clinical variables were not controlled in the final data analysis.

### Impact of tuberculosis on HRQL

Overall, active TB disease had significant and encompassing impacts on patients' HRQL. Using the SF-36, Chamla [28] found that, compared to the general population, people with active TB disease scored significantly lower on PF, RP, GH, BP, and VT (p < 0.05), but no significant differences were observed on RE, SF, and MH subscales (p > 0.05). In general, physical health subscales were more affected than mental ones. Dion et. al. [23,24] also found active TB patients scored significantly lower in SF-36 PCS scores, but not in MCS scores, when compared to people with LTBI and those with previously treated TB disease. In terms of health utility outcomes, Dion et. al. found that active TB patients scored significantly lower in VAS (median 92.5 VS. 97.5, p = 0.02) and SG (median 80.0 VS. 90.0, p = 0.002) than others at the baseline assessment. However, no significant difference was observed in EQ-5D scores between active TB patients and others. It is likely that the small sample size and the heterogeneous composition of subjects could have prevented the authors from detecting the small but important differences in the sample. Wang et. al. [27] found that active TB patients reported lower scores (p < 0.01) across all SF-36 subscales than healthy non-TB people, with RP and RE being most affected. Marra et. al. and Guo et. al. [25,26] found that, compared to those with LTBI, people with active TB scored significantly lower at all SF-36 subscales, SF-6D, HUI-2, HUI-3, and VAS. In contrast, SF-36 scores among people with LTBI before the preventative therapy were very similar to the U.S. norm references.

In the study by Marra et. al. [25], Beck-DI scores showed substantial impairment on mental well-being in active TB patients, compared to people with LTBI. However, many aspects of the Beck-DI (such as fatigue) can also be symptoms of TB and might not be necessarily indicative of mental health impairments. Aydin and Ulusahin [31] compared TB patients to COPD patients and found that TB patients had a lower prevalence of depression and anxiety and a lower level of disability, suggested by GHQ-12 and BDQ scores. The authors postulated that the chronic duration of COPD and the older age of the COPD patients may result in a higher prevalence of psychological impairments. Within TB patients, multi-drug resistant TB patients reported the worst disability level, according to BDQ outcomes. Yang et. al. [29] found that pulmonary TB patients reported more psychological symptoms listed in the SCL-90 and a lower degree of social support using SSRS compared to healthy controls. However, SCL-90 scores did not show significant correlation with SSRS scores, which is not consistent with the established relationship between social support and health [46], as discussed by the authors.

The impaired HRQL experienced by TB patients may be a reflection of socio-demographic status (e.g., age, gender, and socio-economic status) and other underlying co-morbid conditions, besides TB and its treatment. A few included studies explored the relationship between socio-demographic features and clinical factors and HRQL in TB patients. In general, the findings were consistent, but some discrepancies existed. Yang et. al. [29] and Nyamatihi et. al. [32] observed that females were more likely to report poorer health than males, especially on mental health problems, such as depression and anxiety. Chamla [28] and Guo et. al. [26] found older people tended to have poorer HRQL than younger ones. But Duyan et. al. [30] did not find significant associations between gender, age and HRQL in TB patients. On the other hand, they [30] found that better HRQL was correlated with higher income, higher education, better housing conditions, better social security, and closer relationships with family members and friends. Some clinical factors that were observed to correlate with poorer HRQL in TB patients include size of pulmonary TB infection, duration of TB disease, reactivation of previous TB infection, number of symptoms before treatment, development of hemoptysis, hospitalization, underlying chronic conditions, anemia, and count of white blood cells before treatment [27,28].

### Effect of anti-tuberculosis treatment on HRQL

Chamla [28], Dhingra and Rajpal [21], and Marra et. al. [25] prospectively measured active TB patients' HRQL at the start, middle, and end of treatment. In the study by Chamla [28], after the anti-TB treatment, significant improvement was observed in all physical health subscales of the SF-36 (PF, RP, BP, and GH, p < 0.05); two mental health subscales, RE and SF (p < 0.05), improved significantly, but not VT and MH (p > 0.05). During the treatment, RP, VT and MH scores decreased after the initial 2 months and but showed overall improvement at the end of the treatment, while all other subscale scores showed gradual increase over the treatment [28]. Dhingra and Rajpal [21] observed a gradual improvement on DR-12 scores in active TB patients over the course of the treatment. Overall, a more identifiable improvement was observed in symptom scores than that in socio-psychological and exercise adaptation scores. Consistently, Marra et. al. [25] also found significant HRQL improvement in active TB patients over the 6 months of treatment, using SF-36 and Beck-DI.

Although anti-TB treatment improved HRQL overall, active TB patients still had poorer HRQL at the end of the treatment compared to the general population or people with LTBI, especially in psychological well-being and social functioning. Chamla [28] observed that, at the end of the treatment, active TB patients still scored significantly lower at RP, VT, and MH subscales compared to general population comparisons. Marra et. al. [25] found that, after the 6 month of treatment, active TB patients scored significantly lower at SF-36 PCS and MCS summary scores compared to people with LTBI. An interesting finding by Marra et. al. [25] is that, after the preventive treatment, MCS scores among people with LTBI decreased significantly, while PCS scores remained unchanged. Pasipanodya et. al. [34] measured HRQL among pulmonary TB patients who completed at least 20 weeks of treatment, using the SGRQ. Compared with those with LTBI, treated TB patients had lower SGRQ scores. Those with better lung functions and/or born in the U.S. (against foreign-born) tended to have better HRQL outcomes. No gender difference was observed in SGRQ scores.

Muniyandi et. al. [35] assessed the HRQL in a sample of previous TB patients one year after successful completion of treatment. 40% of these people reported persistent symptoms, such as breathlessness, cough, chest pain, and occasional fever. The authors calculated three SF-36 component scores: the physical well-being, mental well-being, and social well-being. Based on their results, there was no gender difference on physical well-being score; but females scored much lower at mental and social well-being scores. Compared with younger people, older ones had significantly lower physical and mental well-being scores, but not the social score. They also presented the U.S. general population norms for the three component scores and concluded that TB patients' HRQL returned to normal level one year after the completion of treatment. However, the way of calculating the three SF-36 component scores is not commonly seen in literatures, and the reference regarding the U.S. general population norms provided in the published paper cannot be located.

## Discussion

HRQL has been appreciated as an important health outcome measure in clinical research. We identified 12 original studies where multi-dimensional HRQL was assessed among people with TB disease or infection using structured instruments around the world. We found that TB and its treatment have a significant impact on patients' quality of life from various aspects and this impact tends to persist for a long time even after the successful completion of treatment and the microbiological 'cure' of the disease.

The results suggest that TB disease has a negative and encompassing impact on active TB patients' self-perceived health status in physical, psychological, and social aspects. Overall, the anti-TB treatment showed positive effect on improving patients' HRQL. It appeared that physical health seemed to be more affected by the disease but improved more quickly after the treatment, while the impairment on mental well-being tended to persist for a longer term [21,28]. However, even after the active TB patients successfully completed the treatment and were considered microbiologically 'cured', their HRQL remained poor as compared to the general population [23-25,28]. The ongoing HRQL impairment may be partly due to the persistent physical symptoms and residual physiological damages from the disease and/or the treatment. Furthermore, a few qualitative studies [9-14,16-18] have shown that the social stigma attached to the diagnosis of TB in some cultures is significant. People with TB may feel isolated from their family and friends or experience the fear and anxiety of being known by others about their diagnosis. All these consequential impairments also need to be 'cured' and may take a long recovery time.

Most studies have focused on assessing HRQL in active TB patients. Although people with LTBI do not present with clinical disease or symptoms, they are likely to be subjected to the same social and psychological impacts as active TB patients. The knowledge of a deadly and stigmatized disease lying dormant in his/her body may also induce anxiety and fear. As Marra et. al. [25] observed that, after receiving 6 months of preventive therapy with isoniazid, the mental well-being of people with LTBI decreased significantly.

HRQL assessment in TB research is still a new area, and a valid and reliable TB-specific instrument is much needed. Currently, a wide range of HRQL instruments were utilized in the literature. The SF-36 was the most frequently used instrument and it appeared to be a valid and reliable tool to be used in TB. Although the SF-36 has been used extensively to assess both population health and specific health conditions for various medical conditions, as a generic health assessment instrument, it offers little information to help understand the unique experiences among TB patients, such as social stigma and anti-TB treatment related ADRs.

Our review identified one TB-specific HRQL instrument, DR-12, which was developed in India [20,21]. Unfortunately, its validation study was not conducted in a systematic fashion and the current evidence provided was not convincing. Further applications and appropriate methodologies are needed to show DR-12 is a psychometrically sound HRQL instrument feasible and valid for TB patients. In addition, the DR-12 is actually designed specifically for pulmonary TB patients, judging from its item content. TB can affect almost any part of the human body, and in Canada, about 40% of active TB diseases would present as extra-pulmonary TB [47]. Different types of TB disease would have very different clinical presentations and affect people's function differently. This may be a challenge when developing a TB-specific HRQL instrument.

It should be also noted that most TB patients have very different cultural and socio-demographic backgrounds compared with the population in which many of these instruments were originally developed. Also, in the studies done in Canada and the USA [24-26,32,34], most TB patients were foreign-born and the instruments were normally self-administered in the English language which would not have been the respondents' first language. Thus, the results of these studies may not be valid if careful translation and cultural adaptation of the instrument was not done to accommodate the multi-cultural population.

Particular attention should be given to some methodological issues on assessing HRQL among people with active TB disease or LTBI. To comprehensively examine the impact of TB and its treatment on patients' HRQL, it is very important to include a proper comparison group from a similar demographic and socio-economic background. When conducting the study, researchers are recommended to seek statistical consultation regarding proper sample size estimating, missing data handling, and adjusting for potential confounders, such as socio-demographic status and presence of co-morbidities. Another concern is the lack of interpretation of HRQL outcomes in terms of clinical meaningfulness. Statistical significance is a useful way to interpret the result, but it fails to relate the HRQL outcome with clinical relevance. As such, more work needs to be done to relate changes in HRQL assessment in TB to concepts such as the minimal clinical difference [48,49].

## Conclusion

Our review of the literature shows that TB diminishes patients' HRQL, as measured by various instruments. However, due to the heterogeneity of HRQL measurements, it was difficult to assimilate results across studies. A few studies used the SF-36 which appeared to be a valid instrument in the measurement of HRQL in TB. Other instruments require further psychometric testing to determine their suitability in measurement in this context. Our review suggests that HRQL assessment in people with TB is a growing research area and a psychometrically sound TB-specific HRQL instrument is lacking. A critical step in the future would be to design an applicable, reliable, and valid TB-specific HRQL instrument. Particular attention should be given to address the methodological issues when conducting a HRQL assessment study in TB patients.

## Competing interests

The authors declare that they have no competing interests.

## Authors' contributions

All authors contributed to the conception and design of the review. NG acquired and analyzed the data and drafted the manuscript. CAM and FM contributed to the analysis and interpretation of the data and finalizing the manuscript. All authors read and gave approval of the final manuscript.

## Supplementary Material

Additional file 1 **Table 1.** Overview of included studies Click here for file

Additional file 2 **Table 2.** HRQL instruments used by included studies Click here for file
