# Supplementary material for: Measuring health-related quality of life in tuberculosis: a systematic review
Source: Health Qual Life Outcomes. 2009 Feb 18;7:14. doi: 10.1186/1477-7525-7-14 (PMC2651863; doi:10.1186/1477-7525-7-14)
Supplement: Additional file 1 — Table 1. Overview of included studies [file 1477-7525-7-14-S1.doc]

*Table 1*. Overview of included studies

| **Reference** | **Publication Year** | **Study Design** | **Study Location**  **and year** | **Patients** | **Comparison group** | **HRQL assessment** |
| --- | --- | --- | --- | --- | --- | --- |
| 21 | 2005 | Prospective cohort | India (2002) | 76 pulmonary or extra-pulmonary TB patients on DOTS | No. | DR-12; at baseline, 2 months and the end of treatment |
| 23,24 | 2002, 2004 | Prospective cohort | Montreal, Canada  (1999-2000) | 46-50 patients (mixture of active TB, latent TB and previously treated TB) | Normative data for Canadian population used as reference. | SF-36 (English and French version), EQ-5D, VAS and SG; at 0, 1 week and 2 weeks |
| 25,26 | 2008 | Prospective cohort | Vancouver, Canada  (2005-2006) | 84 active TB patients for the cross-sectional study; 75-85 active TB patients for the longitudinal study | 78 latent TB patients for the cross-sectional study; 70-75 latent TB patients for the longitudinal study | SF-36 (and SF-6D), HUI-2, HUI-3,  Beck-DI and VAS; at baseline, 3 months and completion of treatment |
| 27 | 1998 | Cross-sectional | China (1996) | 228 hospitalized pulmonary TB patients | 228 healthy controls (matched for age and sex). | SF-36 (Chinese version) |
| 28 | 2004 | Prospective cohort | China (2001-2002) | 102 newly diagnosed TB patients | 103 non-TB controls from the general population (matched for age and sex). | SF-36 (Chinese version); at baseline, 2 months and the end of treatment |
| 29 | 2003 | Cross-sectional | China (2001) | 132 registered pulmonary TB patients | 71 healthy controls | SCL-90 and SSRS |
| 30 | 2005 | Cross-sectional | Turkey (2003-2004) | 120 TB patients hospitalized for at least 1 month | No. | A 24-item QLQ |
| 31 | 2001 | Cross-sectional | Turkey (1999) | 42 newly diagnosed TB, 38 defaulted TB, and 39 multi-drug resistant TB patients | 38 COPD patients (chronic bronchitis and emphysema) | GHQ-12 and BDQ |
| 32 | 2005 | RCT baseline  HRQL assessment | Los Angeles, USA  (1997-2002) | 415 homeless adults with latent TB infection enrolled in a TB-adherence trial | No. | Multiple instruments and questions |
| 33 | 2005 | Cross-sectional | China and Thailand | 84 pulmonary TB patients (52 from China, 32 from Thailand) | No. | SF-36 (Chinese and Thai language version) |
| 34 | 2007 | Cross-sectional | Texas, USA  (2005-2006) | 105 pulmonary TB patients who completed at least 20 weeks of treatment | 207 people with latent TB infection. | MOS core questionnaire and SGRQ |
| 35 | 2007 | Cross-sectional | India | 436 TB patients were assessed one year after they successfully completed treatment and cured | No. | SF-36 |
