# Supplementary material for: Measuring health-related quality of life in tuberculosis: a systematic review
Source: Health Qual Life Outcomes. 2009 Feb 18;7:14. doi: 10.1186/1477-7525-7-14 (PMC2651863; doi:10.1186/1477-7525-7-14)
Supplement: Additional file 2 — Table 2. HRQL instruments used by included studies [file 1477-7525-7-14-S2.doc]

*Table 2*. HRQL instruments used by included studies

| **HRQL Instruments** | **References** |
| --- | --- |
| **Generic** |  |
| Short-Form 36 (SF-36) | 23-28,33,35 |
| MOS core questionnaire | 34 |
| A 24-item Quality of Life Questionnaire (QLQ) | 30 |
| **Specific** |  |
| DR-12 | 20,21 |
| St. George Respiratory Questionnaire (SGRQ) | 34 |
| Symptoms Checklist 90 (SCL-90) | 29 |
| Social Support Rating Scale (SSRS) | 29 |
| General Health Questionnaire 12 (GHQ-12) | 31 |
| Brief Disability Questionnaire (BDQ) | 31 |
| Beck Depression Inventory (Beck-DI) | 25,26 |
| Mental Health Index (MHI-5) | 32 |
| Center for Epidemiological Studies Depression Scale (CES-D) | 32 |
| **Health utility** |  |
| Health Utility Index – 2 & -3 (HUI-2 & -3) | 25,26 |
| Short-Form 6D (SF-6D) | 25,26 |
| EuroQol (EQ-5D) | 23,24 |
| Visual Analogue Scale (VAS) | 23-26 |
| Standard Gamble (SG) | 23,24 |
